# Reply to Wood and Lee, “Precedence for the Role of Indole with Pathogens”

**DOI:** 10.1128/mBio.01787-19

**Published:** 2019-07-30

**Authors:** Vanessa Sperandio, Aman Kumar

**Affiliations:** a Department of Microbiology, UT Southwestern Medical Center, Dallas Texas, USA; b Department of Biochemistry, UT Southwestern Medical Center, Dallas Texas, USA

**Keywords:** CpxA, EHEC, indole

## REPLY

We start by being thankful for the opportunity to respond to the letter by Wood and Lee (1) and clarify several points. We strive to cite the most pertinent literature, although one also has to deal with space constraints. Hence, in several instances, reviews were cited instead of primary literature (e.g., we cited the work of Stevens and Frankel [2] instead of the whole primary literature characterizing EHEC’s type 3 secretion system [T3SS] and attaching and effacing [AE] lesion formation). We also report in our paper (3) that indole was first recognized as a signal by Wang et al. (4), not by Lee at al. (5, 6) as stated by Drs. Wood and Lee in their letter. We also note that we cited the report of Bansal et al. (7) with regard to indole’s role in signaling to mammalian cells and fortifying barrier function.

Drs. Wood and Lee wrote,

“With regard to the mechanism reported by Kumar and Sperandio for sensing indole through the histidine kinase sensor CpxA of EHEC (3), it is important to note that this was discovered 14 years earlier for E. coli by Hirakawa et al. (8). These researchers found that indole sensing in commensal E. coli requires the BaeSR and CpxAR two-component systems. Unfortunately, this contribution was not cited by Kumar and Sperandio (3) [our reference numbers].”

In their paper, Hirakawa et al. (8) state,

“… on the other hand, the induction of *acrD* and *mdtA* was mediated by BaeSR and CpxAR, two component systems. Interestingly, CpxAR system-mediated induction required intrinsic *baeSR* genes, whereas BaeSR mediated induction was observed in the *cpxAR* gene deletion mutant.”

In their paper, Hirakawa et al. (8) show in Fig. 2 that indole increases the expression of both *acrD* and *mdtA* in the Δ*cpxA* and Δ*cpxAR* mutants. We also note that in their studies, Hirakawa et al. employed a concentration of 2 mM indole. As published in our paper (3),

“The CpxAR system is known to be activated by envelope perturbations; hence, at high toxic indole levels (2 mM), an E. coli *cpxR* mutant is responsive to indole, because of perturbations of membrane integrity (9).”

We show genetically and biochemically that CpxA is a sensor for indole, using an indole concentration (500 μM) that does not affect growth or perturb membrane integrity. In Fig. 4 and S5 of our paper (3), we show that the Δ*cpxA* and Δ*cpxA tnaA* mutants do not respond to indole to decrease LEE expression (by quantitative reverse transcription-PCR [qRT-PCR] and Western blotting). We also show that autophosphorylation of CpxA reconstituted in liposomes is decreased in the presence of indole, and this phenotype is not responsive to tryptophan (3).

Drs. Wood and Lee claim the following:

“… it is already established that indole reduces EHEC virulence, in that we showed 12 years earlier that indole repels EHEC (negative chemotaxis), reduces EHEC biofilm formation (a virulence trait), reduces EHEC motility, and reduces EHEC attachment to HeLa cells (a virulence trait) (10).”

However, in the work of Bansal et al. (10), all experiments were conducted with EHEC being grown in Luria broth (LB), a medium that is not conducive to expression of EHEC’s virulence genes *in vitro* (11, 12), as is reflected in their transcriptomic studies, where none of the locus of enterocyte effacement (LEE) or Shiga toxin genes showed up. The LEE and Shiga toxins are the major virulence factors for EHEC (2). Biofilm formation also does not play a role in EHEC’s gastrointestinal disease. Biofilms are important for infection by enteroaggregative E. coli (EAEC), which is a completely different pathovar from EHEC O157:H7 (13). Concerning confusion among pathovars, we point out that in the introduction of the Bansal et al. paper (10), the authors cite Giron et al.’s paper (14) where they state, “An EHEC *luxS* mutant that is deficient in the synthesis of AI-2 and AI-3 demonstrated markedly decreased expression of flagella and motility genes required for adherence to epithelial cells….” Giron et al. (14) describe in their paper that EPEC (not EHEC) has a unique flagellin (H6) that serves as an adhesin to epithelial cells. In Fig. 3 of their paper, Giron et al. show that the EPEC H6 flagellin, but not the EHEC H7 flagellin, binds to epithelial cells. EHEC’s virulence *in vitro* is assessed primarily by expression of the LEE and Shiga toxin and by its ability to secrete T3SS effectors and form AE lesions on epithelial cells; none of these phenotypes were investigated by Bansal et al. (10). We also point out that Citrobacter rodentium, which is extensively used as a surrogate murine infection model for EHEC in the field (15–17), is nonmotile, does not express flagella (18), and is highly virulent.

Drs. Wood and Lee also state, “Indole has also been shown to attenuate the pathogenicity of Staphylococcus aureus (19). Furthermore, indole has been shown previously to act as a true signal for E. coli (20).” On the first point, our paper deals with EHEC and C. rodentium using indole as a signal in the intestine; we are not sure how S. aureus fits into this scenario. On the second point, as stated above, we also cite in our paper that indole was first recognized as a signal by Wang et al. (4), and this was in E. coli.

Drs. Wood and Lee state,

“… it has been argued by us that indole is likely hydroxylated by oxygenases to become an even more potent signal in the gastrointestinal tract (20). Since commensal E. coli produces so much indole in the gastrointestinal tract, we have speculated that indole is the likely archetype for human hormones (6).”

In the paper by Lee et al. (20), all data have to do with biofilm formation on abiotic surfaces; there are no data investigating indole signaling in the gastrointestinal tract. Again, in another paper by Lee et al. (6), the data presented have to do with biofilms on abiotic surfaces and acid resistance *in vitro*. We also point out that the title of the paper by Lee et al. (6) is “Indole Is an Inter-species Biofilm Signal Mediated by SdiA.” The role of SdiA in sensing indole has been deemed controversial (21).

Drs. Wood and Lee state,

“Furthermore, Kumar and Sperandio also failed to indicate that indole has been shown to reduce the virulence of Pseudomonas aeruginosa, another gastrointestinal tract pathogen, by decreasing its *Pseudomonas* quinolone signal (PQS), pyocyanin, rhamnolipid, and pyoverdine production (5).”

Again, we are uncertain how this is pertinent to our paper, which focuses on gastroenteritis by EHEC and C. rodentium (3). In their paper, Lee et al. (5) investigate the role of 7-hydroxyindole (7HI) using a guinea pig aerosol model (Fig. 4) in which they pretreat P. aeruginosa with 7HI and infect the animals by aerosol to assess lung colonization. We also point out that it is hard to assess whether there were statistically relevant differences in levels of infection between the 7HI-treated and nontreated strains. The power was low, with only 5 animals used per group, and the CFU lung counts have differences of 1 × 10^4^ versus 2 × 10^4^.

Drs. Wood and Lee state, “Indole has also been shown to increase the competitiveness of commensal E. coli with P. aeruginosa by inhibiting its quorum sensing (22).” Again we are confused as to the relevance to our studies.

Drs. Wood and Lee state,

“Kumar and Sperandio concluded that manipulation of indole concentrations in the gastrointestinal tract by pre- or probiotics that produce indole can limit the virulence of enteric pathogens (3); however, the use of indole as an antivirulence compound was suggested before by our group (5, 23), and indole was used successfully to reduce the virulence of P. aeruginosa in guinea pigs (5).”

As stated above and repeated here, in their paper, Lee et al. (5) investigate the role of 7HI using a guinea pig aerosol model (Fig. 4) in which they pretreat P. aeruginosa with 7HI and infect the animals by aerosol to assess lung colonization. We also point out that it is hard to assess whether there were statistically relevant differences in levels of infection between the 7HI-treated and nontreated strains. The power was low, with only 5 animals used per group, and the CFU lung counts have differences of 1 × 10^4^ versus 2 × 10^4^. The paper of Lee et al. (23) is a review article.

Drs. Wood and Lee state,

“Hence, Kumar and Sperandio are not the first to show that indole reduces EHEC pathogenicity, not the first to indicate indole is sensed via CpxAR, and not the first to show the importance of indole with non-E. coli strains (both pathogens and nonpathogens).”

Our only claim of a “first” discovery is the measurement of indole concentrations in the intestinal lumen and tissues (we refer to the abstract [3]). These measurements are shown in Fig. 1D and 3. What we show is that indole produced both endogenously (by the engineered C. rodentium strain) (Fig. 5) and exogenously (by a prominent member of the microbiota, Bacteroides thetaiotaomicron) (Fig. 6) decreases C. rodentium virulence gene expression and disease in the gastrointestinal tracts of mice. Moreover, we also show genetically and biochemically (Fig. 4) that CpxA senses indole. None of these data were reported before. To further drive this point home, I am pasting below our abstract with our claims (3).

“Microbial establishment within the gastrointestinal (GI) tract requires surveillance of the gut biogeography. The gut microbiota coordinates behaviors by sensing host- or microbiota-derived signals. Here we show for the first time that microbiota-derived indole is highly prevalent in the lumen compared to the intestinal tissue. This difference in indole concentration plays a key role in modulating virulence gene expression of the enteric pathogens enterohemorrhagic Escherichia coli (EHEC) and Citrobacter rodentium. Indole decreases expression of genes within the locus of enterocyte effacement (LEE) pathogenicity island, which is essential for these pathogens to form attaching and effacing (AE) lesions on enterocytes. We synthetically altered the concentration of indole in the GI tracts of mice by employing mice treated with antibiotics to deplete the microbiota and reconstituted with indole-producing commensal Bacteroides thetaiotaomicron (*B. theta*) or a *B. theta* Δ*tnaA* mutant (does not produce indole) or by engineering an indole-producing C. rodentium strain. This allowed us to assess the role of self-produced versus microbiota-produced indole, and the results show that decreased indole concentrations promote bacterial pathogenesis, while increased levels of indole decrease bacterial virulence gene expression. Moreover, we identified the bacterial membrane-bound histidine sensor kinase (HK) CpxA as an indole sensor. Enteric pathogens sense a gradient of indole concentrations in the gut to probe different niches and successfully establish an infection.”
